# How difficult should it be? Evidence of burden tolerance from a nationally representative sample

**DOI:** 10.1080/14719037.2022.2056910

**Published:** 2022-04-04

**Authors:** Aske Halling, Pamela Herd, Donald Moynihan

**Affiliations:** a Department of Political Science, Aarhus University, Denmark, Europe; b McCourt School of Public Policy, Georgetown University, Washington D.C., USA

**Keywords:** Administrative burden, ideology

## Abstract

There is growing attention to how policymakers and bureaucrats think about administrative burdens, but we know less about public tolerance for burdens. We examine public burden tolerance in two major programmes (Medicaid and SNAP) using a representative sample of US residents. We show broad support for work requirements and weaker support for generally making it difficult to access benefits. People with conservative beliefs, greater opposition to social policies, and higher income are more tolerant of burdens in social policies. Those who have personal experience of welfare policies are less tolerant of burdens.

## Introduction

In recent years there has been growing attention to administrative burdens in policy implementation in many different settings (Carey, Malbon, and Blackwell 2021; Heinrich 2018; Johnson and Kroll 2021; Peeters and Campos 2021). Such burdens are the experience of policy implementation as onerous, and largely emerge from the learning, compliance, and psychological costs people encounter in their interactions with the state (Herd and Moynihan 2018). But while there is a robust and growing literature describing these burdens and their impact on outcomes like programme participation, as well as the political origins of these burdens, we know much less about people's views of these burdens.

In particular, what makes people more or less accepting of administrative burdens, what Bækgaard, Moynihan, and Thomsen (2021) conceptualize as ‘burden tolerance’. Our primary contribution in this paper is to document mass public attitudes towards burdens, where members of the public are asked about specific policies. We study attitudes towards burdens in two of the largest means-tested welfare programmes in the United States: the Supplemental Nutritional Assistance Program (SNAP), better known as food stamps, and Medicaid, which provides health insurance to individuals with lower incomes. While there is varying evidence on burden tolerance in different policy areas, often using experimental designs (e.g. Johnson and Kroll 2021), some of this work focuses on groups other than the public such as policymakers or bureaucrats (Bækgaard, Moynihan, and Thomsen 2021; Bell et al. 2021), or examines the effects of burdens on policy support, rather than the basis of burden tolerance (Keiser and Miller 2020). We know much less about support for burdens based on representative samples, as we employ here.

The question of why the public is more or less tolerant of burdens has high practical salience for three reasons. First, burdens are a central feature in political decisions about policy implementation. In cases when policymakers are reluctant to directly cut the generosity or limit eligibility of welfare policies, they can turn instead to administrative barriers to shrink the policies or make them less accessible. In other words, the implementation of burdens are tied to their politics (Herd and Moynihan 2018). For example, while Donald Trump pledged to protect policies such as Medicaid as a candidate, as President he encouraged states to adopt work requirements in a range of policies where programmatic goals like providing health insurance or improving nutrition were misaligned with these new requirements. By contrast, President Biden has used executive authority to reverse the implementation of work requirements, and directed federal agencies to review ‘systemic barriers in accessing benefits’ in public sector policies and to ‘design experiences with the Federal Government that effectively reduce administrative burdens’.^1^ Second, such burdens can have large effects. The one state that implemented the new Medicaid work requirements for a sustained period of time saw a significant decline in participation, driven by people who were actually working or should have been exempt from the requirement (Sommers et al. 2019). The work requirement therefore excluded people who struggled with the reporting aspects of the process, rather than distinguishing those who were truly eligible from those who were not. Third, political willingness to impose burdens is tied to public beliefs, including indifference, acceptance of their legitimacy, or misunderstanding of their effects. While burdens serve as a form of policymaking by other means, they are most likely employed in settings where there is some public acceptance as to their legitimacy (Fording and Patton 2020). For example, Republican governors turned to work requirements in Medicaid in response to conservative voter beliefs about health insurance becoming too accessible (Fording and Patton 2020).

We survey a nationally representative sample of US residents to better understand the level of support for administrative burdens in specific policy domains, and what factors are correlated with that support. In the absence of verified measures of burden tolerance, we ask both about general impressions of the appropriate level of difficulty to access benefits, plus support for a widely used administrative burden, work requirements.

We find that while members of the US public are split on whether it is too easy or too hard to access benefits, they are more supportive of the specific burden of work requirements. A number of factors associated with tolerance for burdens in these large, redistributive, and means-tested social policies. First, we find that attitudes towards burdens are related to support or opposition for safety net policies in general. While this may seem unsurprising, prior work has called for evidence that distinguishes between attitudes towards safety net policies and burdens as we do here (Bækgaard, Moynihan, and Thomsen 2021, 196). Even controlling for such attitudes, other factors still matter. Second, political ideology predicts burden tolerance, with conservatism associated with support for burdens. Third, people’s experience of the state is associated with their beliefs about burdens: those exposed to welfare policies are less tolerant of burdens. Contrary to expectations, we do not find evidence that people’s self-assessment of their administrative skills is associated with attitudes towards burdens. While our analysis is observational, we assume burden tolerance is causally downstream from other variables, and not subject to reverse causality. In other words, it seems implausible that burden tolerance predicts variables like political ideology for example.

In the next section we summarize the administrative burden literature that we seek to contribute to. Next, we detail how burdens operate in the policy domains we study, and review existing evidence on public attitudes about work requirements in particular. We then propose a series of hypotheses tied to people’s beliefs (support for social policies, political ideology) and experiences (personal experience with the welfare system, and of managing administrative burdens). We then present our data and methods, plus results. Our discussion acknowledges limitations of our analysis, and identifies an agenda for future research.

## Administrative burdens

In this section, we briefly review the administrative burden framework. This framework draws from multiple sources, including red tape research, behavioural science, policy feedback research, and policy studies that centre on the social construction of populations. It takes as a starting point the observation that there is variation in people’s experiences with the state during policy implementation, with some experiences being more onerous than others. Such variation is acknowledged as a possibility, but rarely a central feature of studies of politics or policy, creating a need to better elucidate the nature and sources of these burdens, as well as their impacts on citizens. The framework is broad enough to account for frictions experienced in a wide range of state interactions, such as receipt of social programmes or using individual rights such as voting (Herd and Moynihan 2018), and the experience of citizens in unusual situations such as crises (O’Flynn 2020).

Not all bureaucratic encounters are alike. Moynihan, Herd, and Harvey (2015) argue for demarcating different types of costs that give rise to burdens. Learning costs are the time and effort spent learning about public services, whether they are relevant, and what is involved to engage with them. Lack of awareness of public benefits, or confusion about how to apply, explains why many people do not participate in programmes they would benefit from. Compliance costs are the time and effort involved in applying for or staying on a programme, which might include filling out forms, providing documentation, or satisfying programme requirements. Psychological costs are categories of mental frictions that arise from bureaucratic encounters, which include stress, stigma, frustration, or loss of autonomy.

Some of these costs are driven by factors outside the direct control of high-level policy actors. The discretion that street-level bureaucrats employ may increase burdens for some groups and decrease it for others. For example, welfare case workers are more likely to punish Black welfare recipients than White welfare recipients when they fail to complete a bureaucratic requirement (Schram et al. 2009). Some of the variation in experience may also reflect the human capital and administrative skills that individuals possess (Christensen et al. 2020.; Masood and Nisar 2021; Peeters and Campos 2021).

However, a central claim of the administrative burden framework is that policy designers play a substantive upstream role in enabling burdens. Research on policy take-up shows that hassles can meaningfully limit the reach of the policy, who receives benefits, and the potential of the policy to expand (e.g. Bhargava and Manoli 2015; Fox, Stazyk, and Feng 2020). Burdens are therefore key to policy outcomes, and their existence reflects political and administrative choices: sometimes the choice is to deliberately impose burdens with a good understanding of the effects; sometimes they reflect tolerance of a status quo that goes unquestioned; and sometimes they reflect limited administrative capacity that constrains choices that could reduce burdens, such as the provision of more staff. While they may often be opaque or misperceived by the public, politicians seek to justify burdens, implying that public beliefs matter (Herd and Moynihan 2018). It therefore is useful to better understand public views. The next section explains public discussion of burdens in the two policies that we study.

## Policy background

To understand public views of administrative burdens in particular policy domains, we consider the politics of their use, as well as existing evidence about public support for work requirements in particular. On 10 April 2018, President Trump signed an executive order urging the addition of work requirements for federal welfare policies that did not have them. Trump’s budget proposals also included specific plans to expand work requirements. These plans were largely delayed and frustrated, but reflect an ongoing conservative political policy goal that Trump had championed.

Work requirements have existed in SNAP since 1996, requiring 20 hours of weekly work or training if recipients are to receive more than 3 months of support in a 36-month cycle. States may drop those requirements in cases of economic distress. The Trump administration sought to end this exemption through the rulemaking process. Notably, this rule was developed after legislation to add additional work requirements and other burdens such as drug testing was considered but not included in the 2018 Farm Bill that governs SNAP.

Work requirements were newer to Medicaid, their adoption an example of the spread of burdens into new policies. In the aftermath of Medicaid expansions via the Affordable Care Act (ACA), many states added work requirements. The adoption was driven by conservative politicians who felt pressure to expand Medicaid and capture a new federal source of funding, but who also wanted to demonstrate alignment with conservative constituents who viewed the ACA as an illegitimate form of welfare (Fording and Patton 2019, 2020).

Since Medicaid is an intergovernmental policy, significant changes to the operation of the policy at the state level, such as the addition of work requirements, must be approved by the federal government via a formal request for a waiver from existing policies. The Obama administration denied such waivers, but the Trump administration actively encouraged new waiver requests (Herd and Moynihan 2018). Fifteen states adopted Medicaid work requirements by 2019. These waivers were blocked in court, and in some cases dropped, with the exception of Arkansas which went into effect for long enough that its effects could be studied. The Biden administration quickly rescinded the waivers, rendering moot a Supreme Court case on their constitutionality.

The willingness of Governors to adopt work requirements in Medicaid shows how public beliefs about social welfare policy can influence policy (Brooks and Manza 2007), partly because there is not the same cleavage between citizens with high and low-income on welfare issues that we see in other areas (Gilens 2005). The US public is generally supportive of conditionality for policies that provide support for the poor, including work requirements. A 2013 poll found that 73% of the public supported stricter worker requirements in SNAP in 2013 (Lusk 2013).

A critical insight deriving from the work of Schneider and Ingram (1997) is that burdens are also directed towards populations seen as undeserving, with race often serving as a proxy for deservingness (Michener 2019). In one survey experiment, people were presented with a description of a programme and asked what groups they thought the programme served. When presented with a programme featuring work requirements, people were more likely to assume that such burdens would filter out Black people and immigrants (Haselswerdt 2020). In another survey experiment, White subjects were more likely to tolerate burdensome processes in vignettes with White administrators and Black recipients (Johnson and Kroll 2021). Beneficiaries of policies like SNAP and Medicaid are mostly able-bodied adults, and generally therefore seen as less deserving than, for example, the disabled or elderly (Soss, Fording, and Schram 2011). Medicaid has come to be seen as a form of welfare rather than a health insurance programme, and therefore vulnerable to welfare framing, especially among conservatives (Fording and Patton 2019).

Public support for specific burdens does not mean that public beliefs about them are accurate. Indeed, politicians may exploit public misunderstanding of the scale of effects of burdens, which groups lose out because of burdens, and why they do so (Herd and Moynihan 2018). Public support for work requirements weakens if they are unaccompanied by help, such as child care and transportation (Haeder, Sylvester, and Callaghan 2020). The public also does not fully understand the consequences of work requirements, or at least support the most extreme consequences. Underhill et al. (2020) find people were generally supportive of work requirements for Medicaid, but oppose removing Medicaid if clients do not satisfy these requirements, and proposed lower hour thresholds than was typical of policymakers. In short, people like the idea of a stick, but not actually using it. In the next section, we explain why state actions like work requirements serve as burdens.

### Work requirements as burdens

While the largest and least burdensome US social welfare policies, Social Security and Medicare, are linked to employment, there are three reasons to consider work requirements in means-tested policies like Medicaid and SNAP as a burden, one that is not justified by other policy goals, and which hurt the most vulnerable. While the public may not understand such nuances, they provide the backdrop for public support for administrative burdens.

First, from a purely definitional perspective, burdens are defined as costs experienced in the process of policy implementation (Herd and Moynihan 2018). Demands such as work requirements clearly satisfy this standard, and have been framed as administrative burdens elsewhere (Bækgaard, Moynihan, and Thomsen 2021). They compel the individual to learn about the requirement, establish if the requirement applies to them, and determine how to satisfy the requirement. The requirement creates compliance costs of collecting and reporting appropriate documentation. They may also create a psychological cost arising from seeking such documentation from employers or customers.

Second, the administrative burden framework tends to focus on the adverse effects of burdens. The primary functional effects of work requirements are to exclude large numbers of eligible recipients, rather than sorting out those who are truly eligible versus those who are not. One basic reason why this is the case for policies like SNAP and Medicaid is that most recipients are already seeking work, meaning there are few truly non-eligible people to exclude (Dean, Bolen, and Keith-Jennings 2018; Garfield et al. 2019). A review using longitudinal state administrative data found that work requirements reduced SNAP participation by 52% (Gray et al. 2021). The introduction of work requirements for Arkansas Medicaid recipients reduced coverage from 70.5% to 63.7% of the eligible population, even though 95% of those who lost benefits were actually completing the required work or should have qualified for an exemption (Sommers et al. 2019). The problem was that clients were unaware of the policy or could not overcome the onerous reporting processes. Studies of the effects of work requirements in SNAP (Gray et al. 2021; Han 2022) and Medicaid (Sommers et al. 2019) show no to minimal effect in encouraging labour force participation for the reason cited above: most who can work are working.

Third, work requirements share a characteristic of many other types of administrative burdens in that they exert a disproportionate effect on the most vulnerable groups who also tend to be the most in need of state support. Such groups often lack the skills or supportive environment to manage the burdens (Christensen et al. 2020). For example, those lacking easy access to stable housing, the internet, or human capital skills will struggle more with the requirements. Thus, SNAP work requirements tend to be most effective at excluding the homeless and the lowest income groups (Gray et al. 2021). Work reporting requirements also rigidly demand a level of constancy of work that does not reflect actual work patterns in sectors such as the service industry, or those dependent on seasonal work (Garfield et al. 2018; Schneider and Harknett 2019). Such patterns make it likely that work requirements reduce social equity.

## Beliefs and experiences associated with burden tolerance

Our hypotheses are relatively straightforward and intuitive, and flow from the policy background described above, as well the existing literature on public beliefs about work requirements in particular.

### Beliefs

We first propose that general beliefs about the social safety net will shape views about burdens. This may seem axiomatic, implying that people conceive of policies as abstract bundles of characteristics, where the administrative characteristics are one part of the bundle. If someone dislikes the policy, they should then be more supportive of burdens that make the policy more onerous to access (Herd and Moynihan 2018). For example, people who think Medicaid benefits should be a time-limited are more supportive of work requirements (Haeder, Sylvester, and Callaghan 2020). Thus, we expect a positive correlation between opposition to social policies and tolerance for burdens in social policies.

The relationship between support for policies and support for burdens embedded in those policies is worth parsing for a number of reasons. Bækgaard, Moynihan, and Thomsen (2021, 196) propose that: ‘ … we have limited empirical evidence of how policy support and burden tolerance are related. Future work should better establish rather than assume this relationship, including if, and under what conditions, policy beliefs mediate the effects of other variables such as political ideology on burden tolerance’. It is only through empirical investigation that we can verify if burden tolerance aligns with opposition to the policy, and understand potential nuances in the actual relationship. We can also resolve the question of whether the two variables are essentially identical. Political scientists who have long studied support for social policies might reasonably propose that burden tolerance would simply be a proxy for this opposition to policies. If that is the case, support for social policies should completely mediate the effects of other variables on burden tolerance. If the relationship is not completely mediated that offers a logic for studying burden tolerance as a variable in its own right, even while support for the policy in question is an important control.

H1: People who express higher opposition to social policies will be more tolerant of burdens in social policies.

We next propose that those with a conservative political ideology will be more supportive of administrative burden in social welfare policies. Such support may stem from conservatives having higher concern about levels of government spending, waste, and fraud (Keiser and Miller 2020), or believing that individuals are responsible for their own lot in life, meaning that state support should be offered sparingly (Bell et al. 2021). Conservative politicians support burdens in welfare policies in Denmark (Bækgaard, Moynihan, and Thomsen 2021), and conservative bureaucrats support burdens in redistributive higher education funding policies in Oklahoma (Bell et al. 2021). The adoption of burdens in welfare policies are associated with conservative politicians at the national and state level (Herd and Moynihan 2018). For example, Fording and Patton (2020) code the stances of 99 Governors on Medicaid work requirements from 2014 onwards, finding that of the 33 who supported the requirements, 29 were Republicans. Conservatives are more apt to support work requirements for Medicaid (Haeder, Sylvester, and Callaghan 2020). A coding of US Medicaid application processes found that they tended to feature greater compliance costs – more questions, and more demands for documentation – in states with unified Republican control (Moynihan, Herd, and Rigby 2016). The findings on ideology are not uniform. Earlier survey work by Pereira and Van Ryzin (1998) of New York residents shows no such relationship between ideology and work requirements. There is also little work that examines public views using representative samples.

H2: Conservatives will be more tolerant of burdens in social policies.

### Experiences

We next consider the role of personal experience. We propose that personal experiences of social policies will make individuals less tolerant of burdens. Such a relationship is consistent with a simple incentive account, where people seek to minimize costs. Prior work shows that family experience of burdens in welfare policies also reduces tolerance for these burdens (Pereira and Van Ryzin 1998), and that Medicaid enrollees tend to be less supportive of work requirements in Medicaid (Underhill et al. 2020).

Our approach is also consistent with a policy feedback logic – the direct experience of policies shape client beliefs about policies, and in our case, how they are implemented. The policy feedback perspective suggests that negative experiences may be about more than self-interest: such experiences teach citizens lessons about the political logics of policies, and their role in that logic. For example, direct experience of social policies educates people about how conditions like work requirements actually function: that they are excessively complex and demanding, psychologically draining, and do not serve their claimed purpose. Consistent with this logic, politicians who had received social benefits were more opposed to burdens in social policies, even ones they did not participate in (Bækgaard, Moynihan, and Thomsen 2021). Therefore, unlike peers without direct experience of negotiating welfare policies, those with direct experience are assumed to be more critical of burdens.

H3: Exposure to social policies will be associated with lower tolerance of burdens in social policies.

We next consider how individual experiences of dealing with bureaucratic processes affect burden tolerance. For example, executive functioning, or cognitive skills that allow us to organize and manage complicated tasks, affects people’s ability to manage administrative tasks (Christensen et al. 2020). Such variation has been referred to as human capital (Christensen et al. 2020), or more specifically administrative literacy (Döring 2021; Döring and Krogh-Madsen 2022) and administrative capital (Masood and Nisar 2021). The underlying point is that some people are simply more able and willing to deal with administrative battles. We seek to understand if an awareness of these skills will affect people’s views of burdens. Put another way: we hypothesize that individual acknowledgement that they have high capacity to deal with administrative burdens makes them more accepting of burdens. The straightforward logic is that a distaste for dealing with routine administrative hassles will translate into opposition to administrative hassles in general. The other possibility is that people do not relate their own difficulties with administrative tasks to issues of policy implementation.

H4: Individuals who struggle with administrative tasks will be less tolerant of burdens

### Demographics

We include a series of demographic variables as controls: income, gender, education, religion, race, and age. While prior work has offered some hypotheses on demographic factors (e.g. Pereira and Van Ryzin 1998), they have not controlled for the effects of demographics conditional on support for policies and direct experience of welfare, which themselves will be related to demographic factors. Thus, we do not make predictions about demographic factors here.

## Data and methods

The data come from an online survey that was conducted between 4–8 January 2020 among a national sample of 4,400 US adults. 3,022 of these completed all questions necessary for our analyses. Respondents complete the surveys online via Qualtrics using desktop computers, laptops, or mobile phones. Morning Consult conducts interviews using a network of survey panel providers. To ensure high quality in selecting panels, Morning Consult uses European Society for Opinion and Marketing Research (ESOMAR) documents that contain a uniform set of 26–28 questions for survey panels on sample sources and recruitment, profiling data, respondent privacy and data security, data quality and validation, and incentives.^2^

Respondents may complete a survey at most once every week (8-day minimum exclusion period). They employ randomized matrix or grid-based questions to reduce potential primacy and recency effects; there are also rigorous quality assurance checks in place, including speed (respondents are removed if they complete the survey in less than 4–5 minutes, which is less than one third of the expected survey length) and an attention task that includes implausible statements in a grid-type question. Those who answer implausibly are removed.

Table 1 contains descriptive statistics about the sample and compares it to the American public. Overall, our sample does a fairly good job of resembling the general public. This is especially true for gender, ideology, and race. However, we do also see some substantial differences between the sample and the American population. In particular, we see that the sample contains more people aged 45–64 and fewer non-evangelical Christians than the American population. Since we use weights for our analyses, we also compare the weighted and unweighted sample. The main thing to note from this comparison is that the weights do their job as they generally make the sample representative of the American public.

**Table 1.** Descriptive statistics and comparison with American public.

	Unweighted Sample	Weighted Sample	American Public	Difference^a^
**Age**				
18–34	23.8%	26.3%	23.2%	3,1
35–44	19.0%	17.4%	12.7%	4,7
45–64	36.2%	34.9%	25.3%	9,6
65+	21.1%	21.4%	16.5%	4,9
**Gender**				
Male	52.1%	50.6%	49.2%	1,4
Female	47.9%	49.4%	50.8%	1,4
**Education**				
Below college	57.5%	64.3%	69.4%	5,1
Bachelor’s degree and above	42.5%	35.7%	30.6%	5,1
**Race^b^**				
White	79.5%	72.1%	68.5%	3,6
Black	8.0%	9.9%	13.8%	3,9
Others (inc. Hispanics)	12.5%	18.1%	17.7%	0,5
**Religion**				
Non-evang. Christian	39.6%	39.3%	45.2%	5,9
Evangelical Christian	27.4%	27.2%	25.4%	1,8
All non-Christian	4.9%	4.9%	5.9%	1
Atheist	5.1%	5.0%	3.1%	1,9
Nothing in particular	23.1%	23.6%	19.8%	3,8
**Ideology**				
Liberal	31.0%	31.9%	32.9%	1
Moderate	26.8%	27.6%	27.0%	0,6
Conservative	42.1%	40.5%	40.1%	0,4

Based on the 3,022 respondents included in the analysis. Population data on age, gender, education, and race are from the 2019 and 2020 American Census. Data on religion is from the 2014 PEW Religious Landscape Study while data on ideology is from the 2020 American National Election Study.

^a^Difference between Weighted sample and American Public.

^b^Only based on the population that has a single race.

We next describe the main variables used for our analyses. Full descriptions of survey questions are available in the appendix, Table A4 .

### Dependent variables

The nascent nature of the administrative burden literature means that there are, as yet, are no standard or validated measures of burden tolerance. Therefore, we use two simple measures of tolerance for burdens. One captures how people view barriers to accessing Medicaid and food stamps (‘It is too easy to get federal benefits like Medicaid and food stamps’), while the other focuses on a specific burden – work requirements – on recipients of those programmes (‘Low-income adults who are able to work should be required to do so in order to receive benefits like Medicaid and food stamps’). The first item can be seen as reflecting a broad passive acceptance of burdens, while the other reflects a more active willingness to impose burden. It is possible that other factors, such as general attitudes about the welfare system, shape the first item, though we seek to control for such attitudes in our model. Both questions are Likert-scaled with four categories that include strongly disagree (1), somewhat disagree (2), somewhat agree (3), and strongly agree (4). The correlations between the two items are high at .65 (see Table A1 in the appendix for correlations between all dependent and independent variables).

### Predictor variables

Belief about social policies is measured via responses to the question: ‘Does the government provide too much support or not enough support for the following groups, or do they provide about the right amount of support?’ The responses are tracked via a three category Likert-scaled question where higher values indicate opposition to social policies. Ideology was measured by asking respondents to rate themselves on a scale from 1 (most liberal) to 7 (most conservative), which were grouped into three categories: Liberals (1–3), moderates (4) and conservatives (5–7).

We measure experience with social policies by asking whether the respondent or anyone in their household are, or have been in the past, enrolled in a range of means-tested programmes: Medicaid, SNAP, TANF, Women Infants and Children Nutrition Program, and the Children’s Health Insurance Program, which is linked to Medicaid. We use all policies, rather than just Medicaid and SNAP, because exposure to any programme may affect your tolerance of burden in that or other policies (Bækgaard, Moynihan, and Thomsen 2021).

Finally, we measure struggles with administrative tasks in two different ways. First, we use two questions that ask respondents whether they have ever forgotten to renew their vehicle registration or have paper mail they plan to read that has been unopened for more than a week. We combine these into one measure that captures if respondents have done neither of those, have done one, or have done both. Second, to tap administrative literacy, we ask if respondents have ever received a government document that they did not understand. Categories are simply yes and no. Neither measure is perfect in capturing all facets of struggles with administrative barriers, as they ask for very specific incidents (see Döring 2021 for a more general and carefully developed scale). But this specificity also improves face validity and reduces the risk that the questions tap into broad undefined constructs.

### Model specification

As both dependent variables are ordinal, the appropriate method for analysing the data is ordered logistic regression. However, for ordered logistic regressions to be valid, all categories of all independent variables must meet the parallel regression assumption (also called the proportional odds assumption) (Brant 1990). This assumption states that the effect of independent variables is consistent across different levels of the dependent variable, i.e. that slopes for the different categories of the dependent variable are parallel. The assumption is important because ordered logistic regression summarizes the effect of independent variables in one regression coefficient rather than a coefficient for each level of the dependent variable. We tested the viability of the assumption by doing Brant tests (Brant 1990) and also by using the test incorporated in Stata’s gologit2 package (Williams 2006). Table A2 in the appendix shows the results of these tests. The main conclusion is that the parallel regression assumption is violated for multiple independent variables across models, including a key predictor in opposition to social policies. We also see that results from the two test are highly similar. We therefore turn to a partial proportional odds model instead (Williams 2006). The key difference from an ordered logistic regression is that this model estimates separate coefficients for variables that violate the parallel regression assumption. For all variables that do not violate the assumption, only one coefficient is estimated and results for these variables are therefore equivalent to those obtained by ordered logistic regression. We use the gologit2 command in Stata to determine which variables violate the assumption and set the p-value at .01. We chose the .01 alpha because the risk of false positives is high with a .05-level when doing more than 80 tests in total. Further, Table A2 in the appendix shows that the gologit2 tests are in general more conservative than the Brant test and we therefore found the .01-level appropriate.

We adjust the analyses using probability weights to correct imbalances between our sample and the population of adult Americans. For purposes of comparison, we also include the analyses with the unweighted sample, which is substantively equivalent for the hypothesized variables (see Table A5 in the appendix).^3^ For each dependent variable, we run two separate models. The first model includes exposure to social policies and our measures of individual capacity to manage administrative barriers, as well as demographic control variables: age, gender, race, education, religion, and income. The second model adds opposition to social policies and ideology. Including these in a second model allows us to test both how they predict burden tolerance, as well as the extent to which they mediate the influence of individual capacity and policy feedback (e.g. having direct experience with the burden in policies like Medicaid). Since some of these variables may be highly correlated, we tested for collinearity in our models. Specifically, we calculated the VIF (Variance Inflation Factor) for all variables.^4^ None of the VIF values exceeded commonly proposed thresholds for high multi-collinearity (O’Brien 2007), which suggests that collinearity is not a problem within our data.

Since we rely on observational data, we observe correlations between variables rather than causal effects. The design of the survey does, however, seek to minimize some of the risks of common source bias (Jakobsen and Jensen 2015). For example, our demographic variables and measures of experiences ask subjects to recall factual questions – participation in a program, dealing with administrative tasks. While subjects might suffer from recall bias, this reduces the risk of social desirability bias.

## Results and discussion

**Figure 1.**
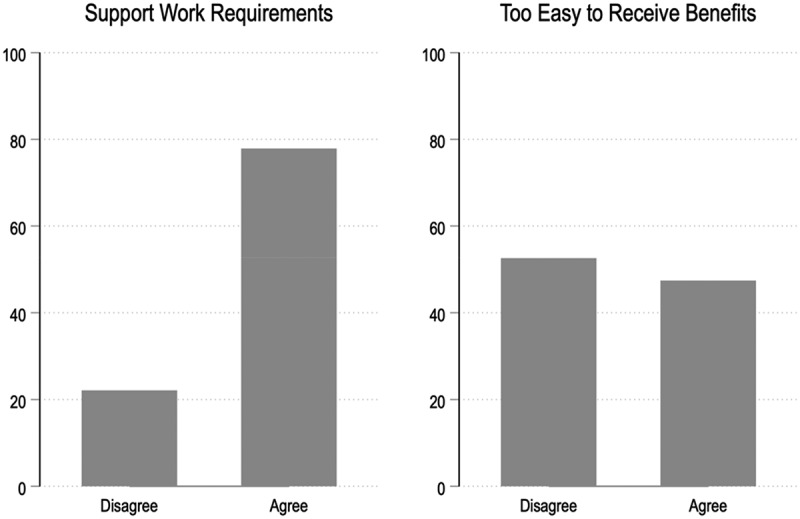
Public views of work requirements and difficulty in accessing benefits.

In simple descriptive terms, there is a basic division in perceptions about how easy or hard it is to get Medicaid or SNAP benefits. Among the respondents who expressed a view, 49% either agreed or strongly agreed that it was too easy to get federal benefits like Medicaid and food stamps, while 51% either somewhat or strongly disagreed. The public expressed stronger support for work requirements: 79% either agreed or strongly agreed that low-income adults who work should be required to do so to receive benefits like Medicaid and food stamps, while 21% disagreed. We exclude the 16% and 12%, respectively, who expressed no opinion from these totals. To simplify the figure, we collapsed the categories ‘strongly disagree’ and ‘somewhat disagree’ into ‘disagree’ while ‘somewhat agree’ and ‘strongly agree’ were collapsed into ‘agree’, as shown in Figure 1.

**Figure 2.**
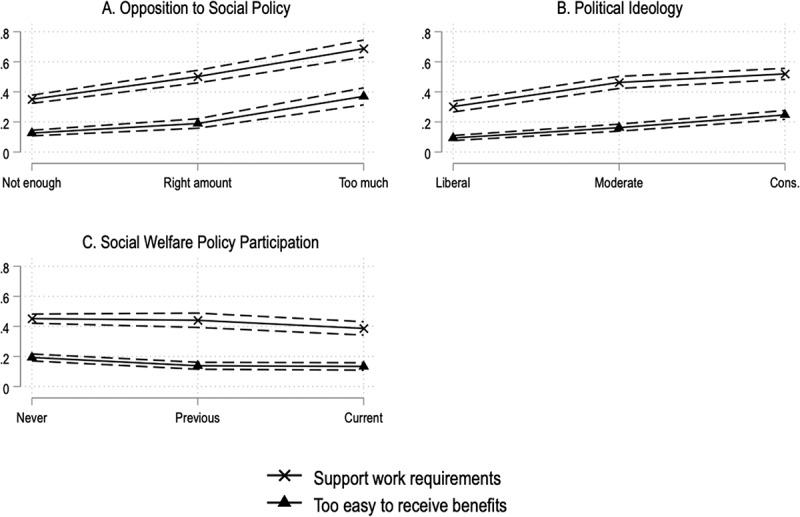
Predicted probabilities for relevant predictors.

The partial proportional odds model presented in Table 2 helps us to understand the basis of these views. In logistic models, coefficients are expressed in log-odds units, which makes substantive interpretation complex. However, the sign (positive/negative) and the statistical significance of the coefficient can be interpreted in a straightforward way. We also present figures depicting predicted probabilities for key predictors in Figure 2. In this figure, we focus on just one category, ‘highly agree’, for both measures of burden tolerance (support for work requirement and views regarding whether social welfare benefits are too easy to access), to ease interpretation. These figures are consistent with the general findings. One descriptive lesson from the figures is relatively robust support for work requirements. Even among Americans who think that the poor receive the right amount of support, half support work requirements (Figure 2a).

**Table 2.** Relationship between opposition to social policies, ideology, social policy participation, individual characteristics, and burden tolerance.

	Work Requirements				Too Easy to Receive Benefits			
	Model 1		Model 2		Model 3		Model 4	
	β	SE	β	SE	β	SE	β	SE
**H1: Opposition to social policy** (ref: Too little support for the poor)								
Right amount of support	–	–	.62**	(.10)	–	–	^a^1.13**	(.16)
	–	–			–	–	^b^-.10	(.15)
	–	–			–	–	-^c^.64**	(.18)
Too much support	–	–	^a^1.11**	(.38)	–	–	^a^1.35**	(.22)
	–	–	^b^.62**	(.23)	–	–	^b^.41*	(.17)
	–	–	^c^.30	(36)	–	–	^c^.05	(.23)
**H2: Ideology** (ref: Liberal)								
Moderate	–	–	.69**	(.11)	–	–	.63**	(.11)
Conservative	–	–	.92**	(.12)	–	–	1.16**	(.11)
**H3: Social policy participation** (ref: never participated)								
Previous participation	−.11	(.11)	−.04	(.11)	−.44**	.10)	−.40**	(.11)
Current participation	−.43**	(.11)	−.27*	(.11)	−.65**	(.11)	−.44**	(.12)
**H4: Failed to renew vehicle registration/unopened mail** (ref: none)								
Done one	−.15	(.09)	−.13	(.10)	−.10	(.09)	−.05	(.10)
Done both	.06	(.17)	.05	(.19)	.13	(.17)	.16	(.17)
**H4: Administrative literacy**	−.13	(.10)	−.12	(.10)	−.11	(.10)	−.08	(.10)
**Observations**	3,022							

Models are estimated using partial proportional odds models. For variables that violate the parallel lines assumption: ^a^ Coefficient for strongly disagree compared to strongly agree (ref), ^b^ Coefficient for somewhat disagree compared to coefficient a, ^c^ Coefficient for somewhat agree compared to coefficient a. Model 2 includes while Model 1 excludes controls for ideology and opposition to social program. The full regression model including all covariates is available in the appendix Table A3

**p < 0.01, * p < 0.05.

The findings support our hypothesis that those who oppose social policies are more supportive of burdens. In Table 2, coefficients for the measure of opposition to social welfare policies were positive and statistically significant: those who believe that the government spends too much on social welfare policies were more supportive of work requirements and were more likely to believe that it was too easy to access social welfare benefits. Figure 2a demonstrates the magnitude of that relationship. The difference in the predicted probability between those who believe that the poor do not get enough support and those who believe they get too much support is .34 for work requirements. This means that, with all other variables held at their mean, an individual opposing social policies is 34 percentage points more likely to highly agree to work requirements in SNAP and Medicaid compared to an individual supporting social policies. The effect for the other item is slightly smaller, with those who express opposition to social policies being 24 percentage points more likely to highly agree that it is too easy for people to receive SNAP and Medicaid benefits. As illustrated by Figure 2, this means that opposition to social policies is the strongest predictor of burden tolerance in our models.

While opposition to or support for social welfare policies have the strongest associations with attitudes about burdens, and mediate the relationship of some other variables, they are not the only factors that matter. Indeed, with the exception of religion and race, adding this variable does not reduce the statistical significance of other predictor variables. Our second hypothesis is that conservatives will be more tolerant of burdens in welfare programmes. We also find strong support of this in our data. As expected, all coefficients for ideology in Table 2, model 2 are positive and statistically significant. Conservatives are 15 percentage points more likely to highly agree that it is too easy to receive benefits, and 22 percentage points more likely to indicate strong support for work requirements. Figure 2b illustrates this relationship. For both outcomes, opposition to social policies is a stronger predictor of burden tolerance than political ideology. While there is obvious overlap between ideology and opposition to social policies – they are correlated at .54 – they are not substitutes when it comes to understanding burden tolerance.

Our next two hypotheses considered the practical experiences people had with welfare policies and administrative tasks, effectively measures of policy feedback. We find support for our third hypothesis that exposure to welfare policies is associated with a lower tolerance of administrative burdens in six of the eight models estimated. Those who are currently receiving welfare benefits have a lower tolerance of burdens than those who never participated in a means-tested welfare programme. This is true for both those who support work requirements and those who think social welfare policies are too easy to access. Those with past participation, compared to those who had no past participation, were less likely to believe that social welfare policies were too easy to access, though there were no differences when it came to views regarding work requirements. This may reflect that work requirements are not present in many of the welfare policies referenced in the survey.

The graph in Figure 2c helps demonstrate the magnitude of these results. Though policy participation is a weaker predictor of burden tolerance than opposition to social policies and ideology, it did have a meaningful association. The differences in predicted probabilities between those who never participated and those currently participating is .06 for the access to benefits question and .07 for work requirements. These results remain robust even after including controls for ideology and opposition to social policies. In short, direct experience of welfare policies is predictive of burden tolerance even when accounting for these important predictors. This finding reiterates the point that support for welfare policies or political ideology does not fully incorporate people’s experiences of welfare when it comes to burden tolerance, and that experiences of US welfare policies generally make people more opposed to burdens. So, for example, a person who is generally conservative and opposed to social spending will become less supportive of barriers in social policies if they have experienced those barriers firsthand. Consistent with the logic of policy feedback, direct experience of the state teaches us lessons about how the state works, and shapes our views about how the state should work.

Our last hypothesis is that individuals who struggle with administrative tasks will be less tolerant of administrative burdens. We do not find support for this hypothesis. We test this with proxies for managing such tasks – did respondents ever forgot to renew their vehicle registration or did they have paper mail which they plan to read that has been unopened for more than a week – and for administrative literacy – did they have trouble understanding government documents. None of the coefficients for these variables are statistically significant in either model in Table 2.

What might explain why people who lack administrative skills are not more opposed to administrative burden, given evidence that the absence of administrative capital matters to accessing benefits (Christensen et al. 2020; Döring and Krogh-Madsen 2022; Masood and Nisar 2021)? The most obvious possibility is that the measures employed do not do an adequate job of capturing the concept, suggesting that better measures might find an effect (Döring 2021). Another possibility is that people do not connect their own administrative skills to their policy views in the way they do with, for example, their political or racial identity. In other words, people might acknowledge personal shortcomings with administrative tasks, but not see how that disadvantages them in their interactions with the state, or that the design of state programmes should consider those shortcomings. Such a possibility is intriguing, since it implies that people do not frame a personal material disadvantage as policy-relevant. We are not aware of any research that has sought to address this question, and so our null finding should be replicated elsewhere.

Our analysis has some limitations. The most obvious is that we rely on a cross-sectional observational design, and associations therefore cannot be considered causal. However, these are also a set of variables where reverse causality appears unlikely, as beliefs about administrative burdens in Medicaid and SNAP are unlikely to drive people’s broader beliefs. We are also able to account for key possible confounders. In particular, we were able to show that the associations between personal experience with social welfare policies and burden tolerance were not simply explained by income, political ideology, or general support for social welfare policies. We are unable, however, to test for deservingness, which has been shown to matter elsewhere (Bækgaard, Moynihan, and Thomsen 2021), and which seems especially salient in the US welfare setting when it comes to beliefs about burdens. On the other hand, the effects of deservingness are likely to be mediated by support for policies. It would also be desirable to distinguish between burden tolerance tied to specific policy domains and burden tolerance more broadly. For example, while the findings on conservatives supporting burdens is consistent with other work, we do not know if the effects of political ideology weaken or even reverse if applied to burdens in other policy domains.

Another limitation is that while we test for whether survey respondents’ race influences their burden tolerance, we’re not able to actually test the influence of racism on these views (Michener 2019). Prior evidence clearly demonstrates that when Black Americans comprise larger proportions of social welfare programmes, people are less supportive of those programmes (Quadagno 1994; Soss et al. 2001; Gilens 2009). Our data, however, do not allow us to test whether this also holds true for burden tolerance, posing an obvious future research question.

A final limitation is the lack of validated measures for some of our items. We use two singleitems to capture burden tolerance. However, even our relatively simple items show high consistency in associations with predictor variables. There is clear value in developing better measures of burden tolerance that can be deployed in different settings, or in using more detailed indicators of skills, such as administrative literacy (Döring 2021).

## Conclusion

A paradox of governance is that people dislike experiencing administrative burdens, but also support imposing them in policies. We find relatively strong support for burdens such as work requirements in means-tested redistributive policies despite evidence that they limit the reach and effectiveness of such programmes (Sommers et al. 2019) and are associated with decreased civic and political engagement (Watson 2015, 645–686). It is therefore substantively important to understand why people tolerate burdens.

We offer evidence on public support and tolerance of burdens in two of the largest US welfare programmes, at a time when there has been increasing political salience in debates about burdens. We show that burden tolerance is partly predicted by support for the policy itself. Those who disagree with welfare policies are more apt to support imposing more requirements that make those policies less accessible. We also offer evidence that while support for a policy and burden tolerance are related, they are not interchangeable. Other factors matter. Consistent with studies of elected officials (Bækgaard, Moynihan, and Thomsen 2021) and bureaucrats (Bell et al. 2021), we find that members of the public with conservative views are more tolerant of burdens in redistributive welfare policies. One implication of our work is that it is worth separating and studying attitudes towards administrative burdens as distinct from attitudes towards social welfare policies generally. We also offer evidence on the importance of personal experience with public policies, or policy feedback effects. While tolerance for burdens reflects general conservative ideology or broader opposition to social welfare policies, we also find evidence that participation in these policies, the actual experience of these burdens, can reduce tolerance for them.

There are many technical solutions to reducing burdens, but the biggest challenge is often the political will to do so. Our results provide insights into the politics, or more specifically why the public tolerates these burdens. One logic for imposing burdens is that they land on the ‘other’. The paradox is that burdens are also universal – anyone can recall a frustrating interaction with a state actor, whether it be a delay renewing a driver’s licence, or confusion with tax returns. While political actors are skilled in framing beliefs by employing political ideology and tropes tied to race or deservingness to increase tolerance of burdens, there is perhaps an untapped opportunity to speak directly to people’s negative experiences of burdens. This again reflects the importance of distinguishing between support for the policy and attitudes about onerous procedures embedded in the policy. Our findings offer evidence that members of the public can make this distinction. This insight may provide clues as to how to alter the politics behind how public organizations are run, as well an impetus for future research to further understand the politics behind public support for burdens.

## Supplementary Material

Supplemental Material

## Notes

1. See Executive Order on Advancing Racial Equity and Support for Underserved Communities Through the Federal Government. https://www.whitehouse.gov/briefing-room/presidential-actions/2021/01/20/executive-order-advancing-racial-equity-and-support-for-underserved-communities-through-the-federal-government/ and Executive Order on Transforming Federal Customer Experience and Service Delivery to Rebuild Trust in Government. https://www.whitehouse.gov/briefing-room/presidential-actions/2021/12/13/executive-order-on-transforming-federal-customer-experience-and-service-delivery-to-rebuild-trust-in-government/

2. The data and code employed can be accessed at https://osf.io/5nrx2/

3. Between the full weighted and unweighted models, the race category of ‘other’ varies in one of the ‘too easy to receive benefits’ model, while income variables change in the support for work requirements models.

4. VIF cannot be calculated based on logistic regression in Stata. Therefore, we rely on ordinary OLS-regression to calculate these.
