# Supplementary material for: How difficult should it be? Evidence of burden tolerance from a nationally representative sample
Source: Public Manag Rev. 2022 Apr 4;25(11):2053–72. doi: 10.1080/14719037.2022.2056910 (PMC10805024; doi:10.1080/14719037.2022.2056910)
Supplement: Supplemental Material [file RPXM_A_2056910_SM9860.docx]

**Appendix**

**Table A1.** Correlations Among Variables

|  |  | (1) | (2) | (3) | (4) | (5) | (6) | (7) | (8) | (9) | (10) | (11) | (12) | (13) |
| --- | --- | --- | --- | --- | --- | --- | --- | --- | --- | --- | --- | --- | --- | --- |
| (1) | Work Requirements | 1.0 |  |  |  |  |  |  |  |  |  |  |  |  |
| (2) | Too Easy to  Receive Benefits | .65 | 1.0 |  |  |  |  |  |  |  |  |  |  |  |
| (3) | Opposition to  Social Programs | .53 | .59 | 1.0 |  |  |  |  |  |  |  |  |  |  |
| (4) | Ideology | .47 | .49 | .55 | 1.0 |  |  |  |  |  |  |  |  |  |
| (5) | Program  Participation | -.18 | -.30 | -.32 | -.15 | 1.0 |  |  |  |  |  |  |  |  |
| (6) | Mail/car | -.11 | -.12 | -.12 | -.14 | .33 | 1.0 |  |  |  |  |  |  |  |
| (7) | Administrative  Literacy | -.09 | -.10 | -.11 | -.07 | .23 | .51 | 1.0 |  |  |  |  |  |  |
| (8) | Age | .12 | .09 | .05 | .18 | -.23 | -.33 | -.18 | 1.0 |  |  |  |  |  |
| (9) | Gender | .01 | -.17 | -.19 | -.15 | .08 | .03 | .01 | -.00 | 1.0 |  |  |  |  |
| (10) | Education | -.06 | .05 | .14 | -.10 | .33 | .03 | -.00 | .06 | -.08 | 1.0 |  |  |  |
| (11) | Religion | .12 | -.14 | -.11 | -.19 | .11 | .10 | .04 | -.19 | -.04 | -.01 | 1.0 |  |  |
| (12) | Ethnicity | -.18 | -.21 | .26 | -.34 | .19 | 22 | -.03 | -.34 | -.07 | -.02 | .13 | 1.0 |  |
| (13) | Income | .11 | .23 | .29 | .05 | -.50 | -.10 | -.11 | .01 | -.12 | .45 | -.06 | -.09 | 1.0 |

Note: Non-weighted Goodman’s Gamma correlations

| **Table A2: Tests for Parallel Lines Assumption** | | | | | | | | |
| --- | --- | --- | --- | --- | --- | --- | --- | --- |
|  | **Work Requirements** | | | | **Too Easy to Receive Benefits** | | | |
|  | Model 1 | | Model 2 | | Model 3 | | Model 4 | |
|  | Brant | Autofit | Brant | Autofit | Brant | Autofit | Brant | Autofit |
| **H1: Opposition to Social Program**  (ref: Too little support for the poor) |  |  |  |  |  |  |  |  |
| Right amount of support | - | - | 0.028 | 0.047 | - | - | 0.001 | 0.001 |
| Too much support | - | - | 0.091 | 0.008 | - | - | 0.020 | 0.008 |
| **H2: Ideology** (ref: Liberal) |  |  |  |  |  |  |  |  |
| Moderate | - | - |  |  | - | - |  |  |
| Conservative | - | - |  |  | - | - | 0.045 | 0.059 |
| **H3: Social program participation**  (ref: never participated) |  |  |  |  |  |  |  |  |
| Previous participation |  |  |  |  |  |  |  |  |
| Current participation |  |  |  |  |  |  | 0.099 | 0.064 |
| **H4: Failed to renew driver’s license/unopened mail** (ref: none) |  |  |  |  |  |  |  |  |
| Done one |  |  |  |  |  |  |  |  |
| Done both |  |  |  |  | 0.098 |  |  |  |
| **H4: Administrative literacy** | 0.078 |  | 0.094 | 0.011 |  |  |  |  |
| **Age** (ref: 18-34) |  |  |  |  |  |  |  |  |
| 35-44 | 0.002 | 0.001 | 0.002 | 0.001 | 0.055 |  | 0.045 |  |
| 45-64 | 0.001 | 0.001 | 0.001 | 0.001 |  |  |  |  |
| 65+ | 0.015 | 0.002 | 0.017 | 0.001 |  |  |  |  |
| **Female** |  |  |  |  |  |  |  |  |
| **Education** (ref: <college) |  |  |  |  |  |  |  |  |
| Bachelor’s degree |  |  |  |  |  |  |  |  |
| Post-grad |  |  |  |  |  |  |  |  |
| **Religion** (ref: non-evang Christian) |  |  |  |  |  |  |  |  |
| Evangelical Christian |  |  |  |  |  | 0.097 |  |  |
| All non-Christian |  |  |  |  |  |  |  |  |
| Atheist |  |  |  |  |  |  |  |  |
| Nothing in particular |  |  |  |  | 0.062 |  | 0.095 |  |
| **Race** (ref: white) |  |  |  |  |  |  |  |  |
| Black | 0.023 | 0.001 | 0.007 | 0.001 | 0.142 |  | 0.079 |  |
| Hispanic |  |  |  |  | 0.057 | 0.032 | 0.050 | 0.039 |
| Other |  |  |  |  |  |  |  |  |
| **Income** (ref: below 20k) |  |  |  |  |  |  |  |  |
| 20k – 50k |  |  |  |  |  |  |  |  |
| 50k – 100k |  |  |  |  |  |  |  |  |
| Over 100k |  |  |  |  |  |  |  |  |
| **Observations** | 3,022 | | | | | | | |
| Note: Entries are p-values and empty cells are p-values > .10 A significant test statistic provides evidence that the parallel lines assumption has been violated. Brant test has been computed based on an unweighted ordered logit regression because Stata cannot do the Brant test on a weighted analysis. Autofit are tests of the parallel lines assumption from the gologit2 Stata add-on with the autofit option. | | | | | | | | |

| **Table A3.** Weighted Relationship Between Opposition to Social Programs, Ideology, Social Program Participation, Individual Characteristics, and Burden Tolerance | | | | | | | | |
| --- | --- | --- | --- | --- | --- | --- | --- | --- |
|  | **Work Requirements** | | | | **Too Easy to Receive Benefits** | | | |
|  | Model 1 | | Model 2 | | Model 3 | | Model 4 | |
|  | β | SE | β | SE | β | SE | β | SE |
| **H1: Opposition to Social Program**  (ref: Too little support for the poor) |  |  |  |  |  |  |  |  |
| Right amount of support | - | - | .62** | (.10) | - | - | ^a^1.13** | (.16) |
|  | - | - |  |  | - | - | ^b^-.10 | (.15) |
|  | - | - |  |  | - | - | -^c^.64** | (.18) |
| Too much support | - | - | ^a^1.11** | (.38) | - | - | ^a^1.35** | (.22) |
|  | - | - | ^b^.62** | (.23) | - | - | ^b^.41* | (.17) |
|  | - | - | ^c^.30 | (36) | - | - | ^c^.05 | (.23) |
| **H2: Ideology** (ref: Liberal) |  |  |  |  |  |  |  |  |
| Moderate | - | - | .69** | (.11) | - | - | .63** | (.11) |
| Conservative | - | - | .92** | (.12) | - | - | 1.16** | (.11) |
| **H3: Social program participation**  (ref: never participated) |  |  |  |  |  |  |  |  |
| Previous participation | -.11 | (.11) | -.04 | (.11) | -.44** | .10) | -.40** | (.11) |
| Current participation | -.43** | (.11) | -.27* | (.11) | -.65** | (.11) | -.44** | (.12) |
| **H4: Failed to renew driver’s license/unopened mail** (ref: none) |  |  |  |  |  |  |  |  |
| Done one | -.15 | (.09) | -.13 | (.10) | -.10 | (.09) | -.05 | (.10) |
| Done both | .06 | (.17) | .05 | (.19) | .13 | (.17) | .16 | (.17) |
| **H4: Administrative literacy** | -.13 | (.10) | -.12 | (.10) | -.11 | (.10) | -.08 | (.10) |
| **Age** (ref: 18-34) |  |  |  |  |  |  |  |  |
| 35-44 | ^a^-.18 | (.21) | ^a^-.19 | (.21) | .11 | (.12) | .16 | (.13) |
|  | ^b^.46** | (.17) | ^b^.51** | (.17) |  |  |  |  |
|  | ^c^.03 | (.21) | ^c^.03 | (.22) |  |  |  |  |
| 45-64 | ^a^.10 | (.20) | ^a^.04 | (.21) | .04 | (.11) | .06 | (.11) |
|  | ^b^.35* | (.16) | ^b^.44* | (.17) |  |  |  |  |
|  | ^c^-.10 | (.20) | ^c^-.05 | (.21) |  |  |  |  |
| 65+ | ^a^.49* | (.24) | ^a^.51* | (.25) | .12 | (.12) | .15 | (.12) |
|  | ^b^.06 | (.21) | ^b^.13 | (.23) |  |  |  |  |
|  | ^c^.-49* | (.24) | ^c^-.52* | (.26) |  |  |  |  |
| **Female** | .03 | (.08) | .21* | (.09) | -.31** | (.08) | -.13 | (.08) |
| **Education** (ref: <college) |  |  |  |  |  |  |  |  |
| Bachelor’s degree | -.18* | (.09) | -.13 | (.09) | .03 | (.09) | .07 | (.09) |
| Post-grad | -.44** | (.11) | -.40* | (.11) | -.31** | (.10) | -.27* | (.10) |
| **Religion** (ref: non-evang Christian) |  |  |  |  |  |  |  |  |
| Evangelical Christian | .39** | (.10) | .19 | (.11) | .40** | (.09) | .12 | (.10) |
| All non-Christian | -.32 | (.18) | -.15 | (.17) | .01 | (.21) | .28 | (.19) |
| Atheist | -.69** | (.17) | -.42* | (.18) | -.54** | (.20) | -.16 | (.20) |
| Nothing in particular | -.35** | (.11) | -.21 | (.12) | -.41** | (.11) | -.26* | (.11) |
| **Race** (ref: white) |  |  |  |  |  |  |  |  |
| Black | ^a^-.73** | (.22) | ^a^-.49* | (.22) | -.59** | (.15) | -.17 | (.16) |
|  | ^b^.54** | (.14) | ^b^.57** | (.14) |  |  |  |  |
|  | ^c^.47* | (.22) | ^c^.57* | (.22) |  |  |  |  |
| Hispanic | -.24 | (.19) | -.08 | (.21) | -.15 | (.20) | .07 | (.20) |
| Other | -.43** | (.15) | -.28 | (.15) | -.29 | (.15) | -.05 | (.16) |
| **Income** (ref: below 20k) |  |  |  |  |  |  |  |  |
| 20k – 50k | .28* | (.14) | .22 | (.15) | .17 | (.13) | .11 | (.14) |
| 50k – 100k | .46** | (.15) | .33* | (.15) | .63** | (.13) | .52** | (.15) |
| Over 100k | .57** | (.16) | .37* | (.17) | .75** | (.15) | .59** | (.16) |
| **Observations** | 3,022 | | | | | | | |
| Note: Models are estimated using partial proportional odds models. For variables that violate the parallel lines assumption: ^a^ Coefficient for strongly disagree agree compared to strongly agree (ref), ^b^ Coefficient for somewhat disagree compared to coefficient a, Coefficient for somewhat agree compared to coefficient a.  Model 2 includes while Model 1 excludes control for ideology and opposition to social program.  ** p<0.01, * p<0.05. | | | | | | | | |

**Table A4.** Survey Questions

| Measure | Question | Scale |
| --- | --- | --- |
| Burden Tolerance | Do you agree or disagree with each of the following?  (1) It is too easy to get federal benefits like Medicaid and food stamps | 1 Strongly disagree  2 Somewhat disagree  3 Somewhat agree  4 Strongly agree |
|  | (2) Low income adults who are able to work should be required to do so in order to receive benefits like Medicaid and food stamps |  |
|  |  |  |
| Opposition to Social Programs | Does the government provide too much support or not enough support for the following groups, or do they provide about the right amount of support?  “Poor people” | 1 Not enough support  2 About the right amount  3 Too much support |
| Ideology | Thinking about politics these days, how would you describe your political viewpoint? | 1 (liberal) –  7 (conservative) |
| Program participation | Are you or someone in your household currently or have ever been previously enrolled in the following programs, or not?  (Medicaid, SNAP, Temporary Assistance for Needy Families, Women Infants and Children Nutrition Program, and The Children’s Health Insurance Program) | 1 No  2 Yes |
| Mail/car | Have any of the following ever expired before you could renew it, or not?  “Your car registration”  Do each of the following describe you or not?  “I have paper mail I plan to read that has been unopened for more than a week” | 1 No  2 Yes |
| Administrative  Literacy | Do each of the following describe you or not? “I have received a government document in the mail that I did not understand” | 1 No  2 Yes |

| **Table A5.** Non-weighted Relationship Between Opposition to Social Programs, Ideology, Social Program Participation, Individual Characteristics, and Burden Tolerance | | | | | | | | |
| --- | --- | --- | --- | --- | --- | --- | --- | --- |
|  | **Work Requirements** | | | | **Too Easy to Receive Benefits** | | | |
|  | Model 1 | | Model 2 | | Model 3 | | Model 4 | |
|  | β | SE | β | SE | β | SE | β | SE |
| **H1: Opposition to Social Program**  (ref: Too little support for the poor) |  |  |  |  |  |  |  |  |
| Right amount of support | - | - | .74** | (.09) | - | - | ^a^1.18** | (.14) |
|  | - | - |  |  | - | - | ^b^-.00 | (.12) |
|  | - | - |  |  | - | - | ^c^.-61** | (.16) |
| Too much support | - | - | ^a^1.61** | (.13) | - | - | ^a^1.35** | (.19) |
|  | - | - | ^b^.62** | (.23) | - | - | ^b^.43** | (.16) |
|  | - | - | ^c^.30 | (36) | - | - | ^c^.15 | (.21) |
| **H2: Ideology** (ref: Liberal) |  |  |  |  |  |  |  |  |
| Moderate | - | - | .73** | (.09) | - | - | .66** | (.09) |
| Conservative | - | - | 1.14** | (.10) | - | - | 1.28** | (.10) |
| **H3: Social program participation**  (ref: never participated) |  |  |  |  |  |  |  |  |
| Previous participation | -.10 | (.10) | -.01 | (.10) | -.48** | (.09) | -.44** | (.09) |
| Current participation | -.50** | (.10) | -.28* | (.10) | -.70** | (.09) | -.61** | (.11) |
| **H4: Failed to renew driver’s license/unopened mail** (ref: none) |  |  |  |  |  |  |  |  |
| Done one | -.13 | (.08) | -.09 | (.08) | -.15 | (.08) | -.09 | (.08) |
| Done both | .03 | (.15) | .02 | (.15) | .05 | (.14) | .08 | (.15) |
| **H4: Administrative literacy** | -.13 | (.08) | -.11 | (.08) | -.11 | (.08) | -.09 | (.08) |
| **Age** (ref: 18-34) |  |  |  |  |  |  |  |  |
| 35-44 | ^a^-.24 | (.19) | ^a^-.27 | (.19) | .00 | (.10) | .03 | (.11) |
|  | ^b^.43** | (.15) | ^b^.46** | (.15) |  |  |  |  |
|  | ^c^.18 | (.19) | ^c^.18 | (.20) |  |  |  |  |
| 45-64 | ^a^.09 | (.17) | ^a^.04 | (.21) | -.04 | (.09) | -.04 | (.09) |
|  | ^b^.34* | (.14) | ^b^.38* | (.15) |  |  |  |  |
|  | ^c^-.08 | (.17) | ^c^-.07 | (.18) |  |  |  |  |
| 65+ | ^a^.38 | (.24) | ^a^.43* | (.22) | .04 | (.10) | .03 | (.11) |
|  | ^b^.12 | (.18) | ^b^.13 | (.19) |  |  |  |  |
|  | ^c^.-33 | (.22) | ^c^-.41 | (.23) |  |  |  |  |
| **Female** | .01 | (.07) | .25** | (.09) | -.41** | (.07) | -.20** | (.07) |
| **Education** (ref: <college) |  |  |  |  |  |  |  |  |
| Bachelor’s degree | -.19* | (.09) | -.12 | (.09) | -.00 | (.08) | .07 | (.09) |
| Post-grad | -.46** | (.10) | -.38** | (.10) | -.31** | (.10) | -.24* | (.10) |
| **Religion** (ref: non-evang Christian) |  |  |  |  |  |  |  |  |
| Evangelical Christian | .40** | (.09) | .14 | (.09) | .42** | (.08) | .12 | (.08) |
| All non-Christian | -.30 | (.16) | -.07 | (.17) | -.04 | (.16) | .26 | (.16) |
| Atheist | -.82** | (.16) | -.40* | (.16) | -.83** | (.16) | -.31 | (.16) |
| Nothing in particular | -.32** | (.09) | -.15 | (.09) | -.46** | (.09) | -.29* | (.09) |
| **Race** (ref: white) |  |  |  |  |  |  |  |  |
| Black | ^a^-.84** | (.19) | ^a^-.55* | (.20) | -.68** | (.13) | -.21  (.13) | |
|  | ^b^.46** | (.14) | ^b^.59** | (.14) |  |  |  |  |
|  | ^c^.47* | (.22) | ^c^.63** | (.21) |  |  |  |  |
| Hispanic | -.26 | (.16) | -.08 | (.17) | -.26 | (.16) | -.05 | (.17) |
| Other | -.37** | (.13) | -.17 | (.13) | -.39** | (.13) | -.15 | (.13) |
| **Income** (ref: below 20k) |  |  |  |  |  |  |  |  |
| 20k – 50k | .16 | (.14) | .09 | (.12) | .21 | (.12) | .15 | (.12) |
| 50k – 100k | .35** | (.12) | .19 | (.12) | .61** | (.12) | .49** | (.12) |
| Over 100k | .49** | (.14) | .24 | (.15) | .75** | (.14) | .55** | (.14) |
| **Observations** | 3,022 | | | | | | | |
| Note: Models are estimated using partial proportional odds models. For variables that violate the parallel lines assumption: ^a^ Coefficient for strongly disagree agree compared to strongly agree (ref), ^b^ Coefficient for somewhat disagree compared to coefficient a, Coefficient for somewhat agree compared to coefficient a.  Model 2 includes while Model 1 excludes control for ideology and opposition to social program.  ** p<0.01, * p<0.05. | | | | | | | | |
